# Using 5TE Sensors for Monitoring Moisture Conditions in Green Parks

**DOI:** 10.3390/s24113479

**Published:** 2024-05-28

**Authors:** Muawia Dafalla

**Affiliations:** Department of Civil Engineering, College of Engineering, King Saud University, Riyadh 11421, Saudi Arabia; mdafalla@ksu.edu.sa

**Keywords:** temperature, heat, clay–sand liners, mixtures, 5TE sensors

## Abstract

The ground surface and subsurface of green parks in arid and desert areas may be subjected to desiccation as a result of weather and hot temperatures. It is not wise to wait until plants are turning pale and yellow before watering is resumed. Given the scarcity of water in typical desert zones, we recommend full control of irrigation water. This study presents a method of recycling irrigation water using 5TE sensors, employing time-domain reflectometry (TDR) technology. A trial test section was constructed along the coast of the eastern province of Saudi Arabia. Water recycling involves using clay–sand liners placed below the top agricultural soils to intercept excess water and direct it towards a collection tank, and then it is pumped out to a major water supply tank. The main properties of soils and clay–sand liners normally taken into account include moisture content, density, and hydraulic conductivity. An assessment of geotechnical properties of clay–sand mixtures containing 20% clay content was conducted. The profiles of moisture and temperature changes were monitored using 5TE sensors and data loggers. The 5TE sensors provided continuous measurements at varying temperatures and watering cycles. Twenty-nine watering cycles were conducted over a six-month period. An additional section was considered with a liner consisting of the same clay but enhanced with bentonite as one-third of the clay content. The volumetric water content was found to vary from 0.150 to 0.565 following changing weather and direct watering cycles. The results indicated that the use of a TDR instrumentation is a cost-effective and time-saving technique to construct a system for saving irrigation water.

## 1. Introduction

The measurement of soil moisture content is of great importance to determine many geotechnical parameters and the water balance in environmental and agricultural projects. So many approaches can be followed to obtain the moisture content. Oven techniques, hot plates, microwaves, and chemical methods were all among the methods used in practice. These methods require time and attendance to perform. The need for immediate and reliable results suggested the use of sensors that can obtain the result in a very short time and can be digitally traced and reported. The most common sensors include a contact-based technique known as time-domain reflectometry (TDR) [1,2]. These sensors were found to be highly accurate and were tested by many investigators for various environmental exposures and soil types [3,4,5].

This study is designed to investigate the use of 5TE moisture sensors in the management of irrigation, wetting, and drying assessments of green parks. This type of sensor measures the volumetric moisture content, temperature, and electrical conductivity. The volumetric moisture content is obtained using the dielectric constant of the media based on capacitance/frequency domain technology. It normally measures the apparent dielectric permittivity to an accuracy of 1 εa for the soil range (1–40), resulting in +/− 3% using the Topp equation. 

The dielectric permittivity is computed by measuring the delay in time between the incident and reflected electromagnetic pulses [6,7]. 

Topp et al. (1980) [5] presented an empirical relationship between the dielectric constant (Ka) and the volumetric moisture content (θ) for soils of variable mineralogy.

*θ* = 4.3 × 10^−4^ K_a_^3−^− 5.5 × 10 ^−4^ K_a_^2^ + 2.92 × 10^−2^ K_a_^−^− 5.3 × 10^−2^

where:

*θ* = volumetric water content
*Ka* = dielectric constant.

Wang et al. [8] investigated the use of the Topp equation for two soil materials and presented an assessment on the accuracy based on the laboratory-measured gravimetric moisture content.

Numerous studies have carried out field calibration, in which the sensor output is compared to the volumetric water content of the field soil, which is determined using the gravimetric approach [9]. However, some researchers [10,11] suggested field calibration for greater accuracy in a laboratory soil calibration conducted by the manufacturer for specified types of soil can be satisfactory. The manuals provided by Meter (2018) provide detailed methods of application and use [12,13].

There are three phases in unsaturated soils: solid, air, and water. According to Shmulik [14], the aqueous solution is the only conducting phase for apparent electrical conductivity. This opened up the possibility of using it for the assessment of volumetric water contents (θ). Nonetheless, a wide range of variables, such as temperature, cation composition, particle shape and orientation, soil density, and porosity, might affect the measurement of electric conductivity. A rise in temperature has the potential to reduce the electrical resistivity of soil.

Studies that examined the effect of temperature include the works of Seyfried et al. and Banon et al. [15,16]. The impact of temperature changes on the moisture content can be clearly observed when the temperature is recorded at 40 °C or higher [17], and this effect is also noticed in the current study extending over six months, where the profile of moisture is different during the hot period from 32 °C to 40 °C. The permittivity measurement is also affected by the salinity of soil, but this effect is practically negligible when the salinity is <1 dS∙m^−1^. 

Understanding the temporal variations in soil temperature is equally important for plants or the cover of vegetation. The ambient temperature and the surrounding environment are significant factors to many plant processes [18]. The following is how Bergman et al. [19] applied Newton’s equation of cooling, which connects the heat transfer coefficient λ to the soil temperature Tsoil:

Tsoil,t = Tenv,t − ∆T exp (−λ)(1)

The clay of Al-Qatif is extensively studied by researchers and was found to have high plasticity and be suitable for constructing liners for environmental and waste-control purposes [20]. In this study, the clay–sand liner is intended for use as a water barrier to intercept excess water penetrating towards deep sand deposits. This local clay is suggested as an alternative to bentonite material. The governing factor for using typical clay is its hydraulic conductivity. It is sometimes likely to fall short of the specified level of hydraulic conductivity but can be enhanced with a small amount of bentonite.

The objective of this paper is to study the use of 5TE sensors linked to a proposed drainage system of liners. This is intended to save water in green parks and monitor moisture variations. Installing this sensor type is practical and can provide continuous data and information that enable quick decisions needed for watering grass and the collection of surplus water using automatic or manually controlled pumps. This research paper is structured to introduce the concept of utilizing the 5TE sensors and data loggers in volumetric water content observations and also to study the impact of temperature, considering wetting and drying cycles. The methodology is given in Section 3. Results and discussions are presented in Section 4. The conclusion of the work and suggestions for future studies are given in Section 5.

## 2. Related Studies

The introduction of sensor technology and data loggers encouraged automation and use of sensors for water management. Most of the recent works are focused on agricultural applications [21,22]. The current study is mainly targeting the influence of environmental and geoenvironmental influence on the subsurface soils. The parameters investigated can also help in water management and plant demand. Balatsouras et al. [23] claimed that frameworks similar to WiChord+ are promising systems due to their compatibility with modern devices and accessories, providing an echosystem for smart agricultural approaches. A significant part of water is lost due to evapotranspiration (ET). Payero [24] suggested a simple weighing method to determine the evapotranspiration, which includes both plant transpiration and water evaporation from the soil surface. It is a vital part of the water cycle and affects the ecosystem’s overall water balance. 

Cariou et al. [25] highlighted the importance of buried sensor nodes and discussed its potential agricultural applications. Bertocco et al. [26] introduced a comparison between three approaches to assess the volumetric water content using ML algorithms. They concluded that an augmented VWC sensing method relying on a received signal strength indicator (RSSI) and soil-moisture sensor reading gave better results. The factors influencing the measurements of the volumetric moisture content are numerous, and there is a lot to investigate in this regard. This study observed the measurements of the volumetric water content at different temperature and watering patterns.

The field work related to this research considered sections of different types of clay–sand liners, and the sensors cover only the sections of concern. However, for vast and extended green areas, multiple sensors can be provided. A wireless sensor network (WSN) is an approach to collect data for extended and remote areas. Zhang et al. [27] claimed that in situ soil moisture observation can be reported in a radius exceeding 20 km. This is mainly dependent on careful selection of the required range. For field irrigation systems, this may not be essential. Few stations within the area may be sufficient to serve the purpose. 

The work presented in this study is a further development that can serve irrigation systems, environmental assessment, and water management. This will certainly advance the current state-of-the-art of using underground sensors.

## 3. Materials and Testing Procedures

### 3.1. Materials

#### 3.1.1. Sand

**Figure 1:**
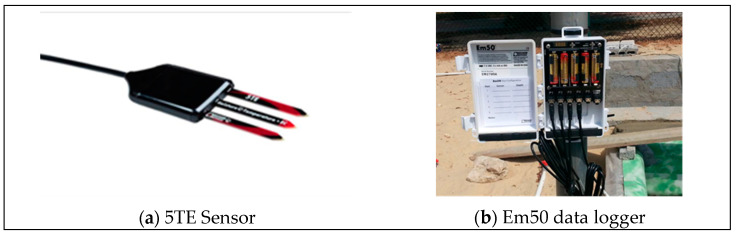
5TE moisture sensor and Em50 data logger.

The primary component of the clay liners is sand. The sand source for this study is a fine- to medium-grained material that is abundant in the nearby desert regions. The unified soil classification system, or ASTM D 2487 [28], is used to classify the sand as SP “poorly graded sand”. The material was found to be outside of the uniformly graded sand range after looking at the coefficients of curvature (1.078) and uniformity (1.737). The sand used has a specific gravity of 2.65 to 2.66. The sand’s particle size distribution is seen in Figure 1. In this study, the sand was used to compose layers for use in liners and layers overlying the liners for green parks. It is placed right under the agricultural soil and on top of the proposed liners to help in the drainage of water towards a collection point.

#### 3.1.2. Natural Clay

The clay used in this study was obtained from Al-Qatif town, located in the eastern province of Saudi Arabia. The specific gravity, soil classification, and index parameters of Al-Qatif clay are shown in Table 1. Al-Qatif clay, which is categorized as CH in the unified soil classification system, or ASTM D 2487 [28], is a highly plastic soil that is well known for its strong expansion and shrinkage capabilities [29,30].

The composition of Al-Qatif clay is shown in Table 2. The ASTM D 698 [31] standard was followed in examining the moisture–density relationship of the clay, which was found in the range of 11.5 to 12 kN/km^3^, but when testing the clay–sand mixtures with 20% clay by weight, the optimum moisture content level was 15 to 17%, and the maximum dry density was 17.5 to 18.00 kN/m^3^.

Sand–clay mixtures were prepared using a clay content of 20%. The compaction properties of these mixtures were measured. 

#### 3.1.3. Bentonite Clay

For this study, the commercial bentonite HY Oil Companies Material Association (OCMA) was chosen. The liquid limit was reported as 480, and the plastic limit was measured as 50, resulting in a plasticity index of 430. The specific gravity was found in the range of 2.6 to 2.7. The chemical components of the HY OCMA bentonite are shown in Table 2. 

### 3.2. Field Sections: Installation and Procedure 

The main idea of this work was to develop a system that can save irrigation water and monitor environmental conditions during the lifetime of a green vegetation cover. All soils were prepared at the maximum dry density and the optimum moisture content, as relevant to the required clay content and the likely set-up. The main section addressed here consisted of 20% Al-Qatif clay liner. This was chosen based on previous studies conducted by the author. It was found that the more clay content, the lower the hydraulic conductivity of the liner. The natural clay of Al-Qatif cannot perform as the commercial bentonite with regard to the water-retention capacity. This is basically attributed to the lower plasticity. One other section was constructed in a similar way, but the natural Al-Qatif clay was enhanced with one-third bentonite (33.3% of the clay). The excavations for each section were 2 m long, 1 m wide, and 0.6 to 0.7 m deep. The material was placed and compacted in layers using small compacting machines to achieve the required thickness and the densities, as relevant to the standard proctor test.

**Figure 2:**
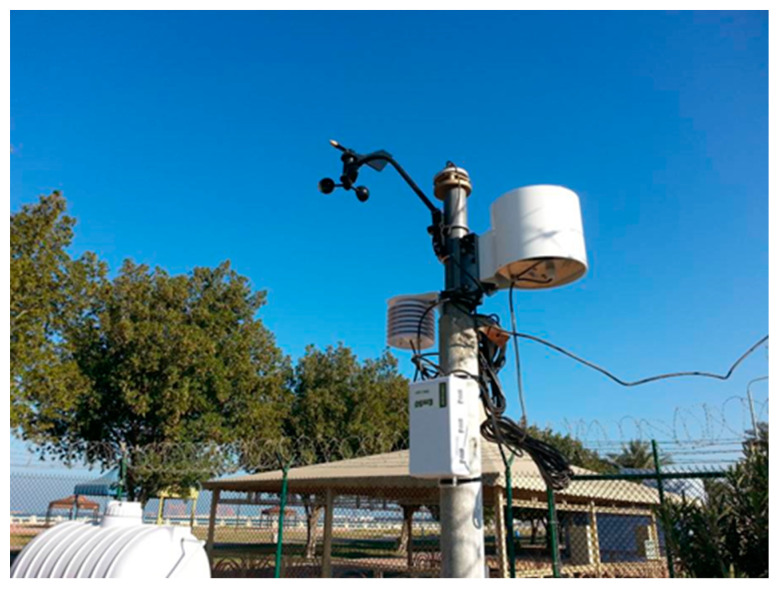
A weather station constructed on site.

**Figure 3:**
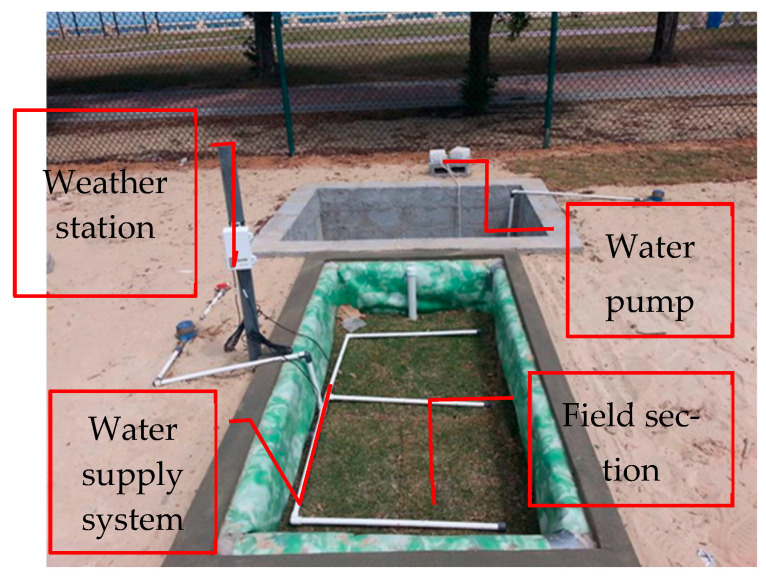
Typical field section.

A weather station was installed to monitor the temperature, rainfall, wind, and other parameters. Figure 2 presents the weather station constructed on site. Figure 3 shows a typical field section.

**Figure 4:**
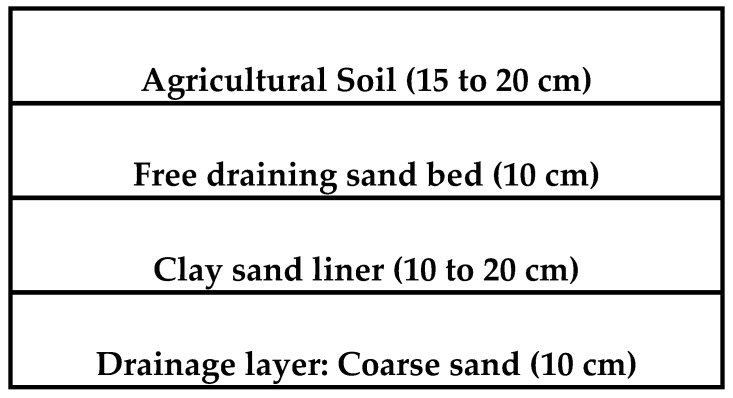
Typical layers of the field section.

Figure 4 shows a sketch of these layers, which are composed of top agricultural soil, mainly silty sand, followed by a sand layer and then the clay–sand liner. The thickness of the liner is 20 cm, and it is also underlain by 10 cm of free draining sand.

Variations in volumetric water content, temperature, and electrical conductivity were recorded using 5TE sensors (Figure 1) connected to an Em50 data logger. In addition to the sensors placed within the clay–sand liner marked as A and B, one more sensor was placed to record the ambient temperature and moisture. Table 3 presents the 5TE sensor specifications.

The sensors were set to take records at one-hour intervals.

**Figure 5:**
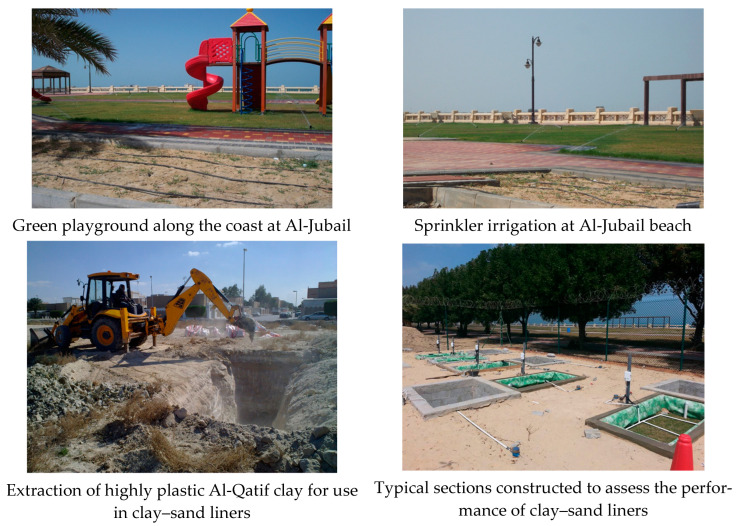
Photographs showing the site and clay material excavation works.

**Figure 6:**
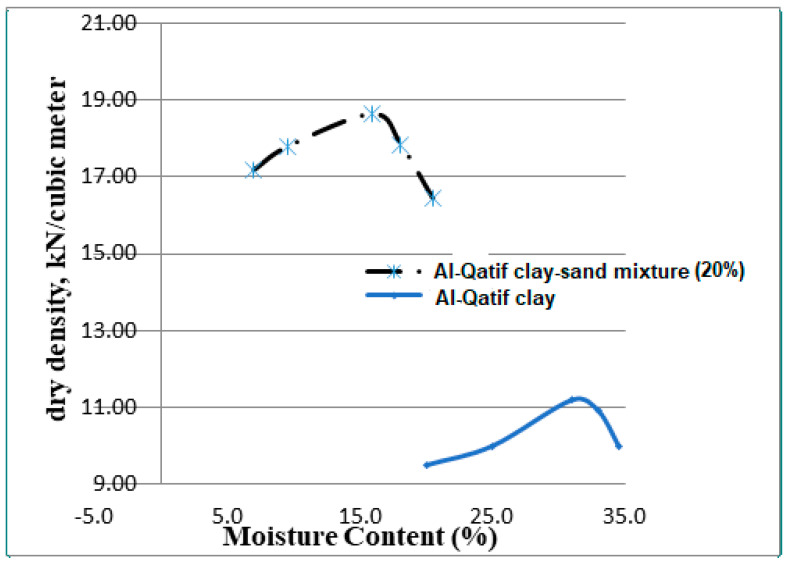
Maximum dry density versus moisture content for the clay–sand mixture with 20% clay and for Al-Qatif clay.

The 5TE sensors were attached to the mid-depth of the clay–sand layer. One more sensor was installed to record the ambient temperature. Figure 5 presents photographs from the site and methods of extracting clay in the Al-Qatif region. Figure 6 presents the maximum dry density versus moisture content for the clay–sand mixture with 20% clay and for Al-Qatif clay.

## 4. Results and Discussion

A system of irrigation control proposed for this research is aimed at collecting excess water resulting from excessive irrigation. This is achieved by intercepting seeping water with the sand–clay liner. The excess water is directed towards an underground water tank. An automatic pump supplied to the underground water tank lifts water to a large on-ground water tank, acting as a main supply to the field sections. In practice, this tank can be eliminated and replaced by slopes of the ground made so that excess water is collected in a large sump or underground tank at one corner or edge of the park. From there, water can be recirculated for irrigation again. After a number of cycles, the water needs to be checked for salinity and suitability for the types of plants or grass used in the park.

The knowledge of the moisture content profile over time is very useful to assess the condition and make the decision to intervene if watering is required. The program of the study included performing 29 direct watering tests applied to a section, with a 20 cm thick clay–sand liner made up of Al-Qatif clay and sand (the clay is 20% by weight of the mixture). The second section considered for this study was for a bentonite-enhanced natural Al-Qatif clay. In this section, one-third of the clay was replaced by commercial bentonite. Similar monitoring was conducted. The hydraulic conductivity of a range of clay-sand mixtures was presented by Dafalla et al. [32]. It can be seen that the hydraulic conductivity is reduced by increasing the clay content. The consideration of bentonite is to achieve a better hydraulic conductivity suitable to retain excess water.

Table 4 provides a summary of 29 direct watering tests conducted in the field with specified dates spanning a six-month period.

The flowmeter readings for water entering the distribution pipes show the amount of water supplied each time. Records associated with each watering test are stored in the data loggers.

**Figure 7:**
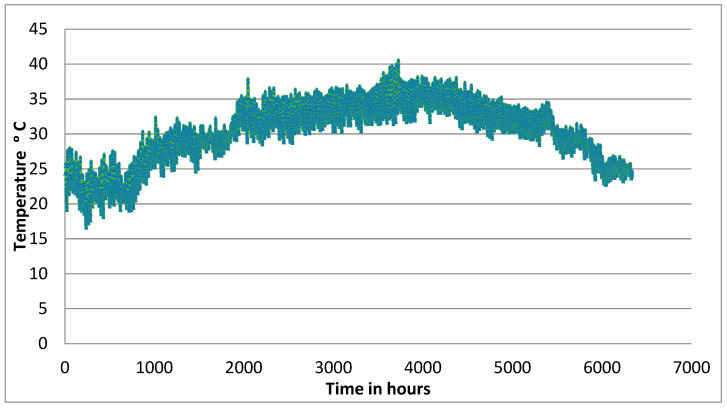
Maximum and minimum daily temperature over 6-month period.

Figure 7 presents the ambient temperature profile for the site for the whole period of six months, from March to November. The range for minimum and maximum daily temperatures was reported in the range of 16 °C to 40 °C. Rainfall was traces, and no records above zero were reported.

**Figure 8:**
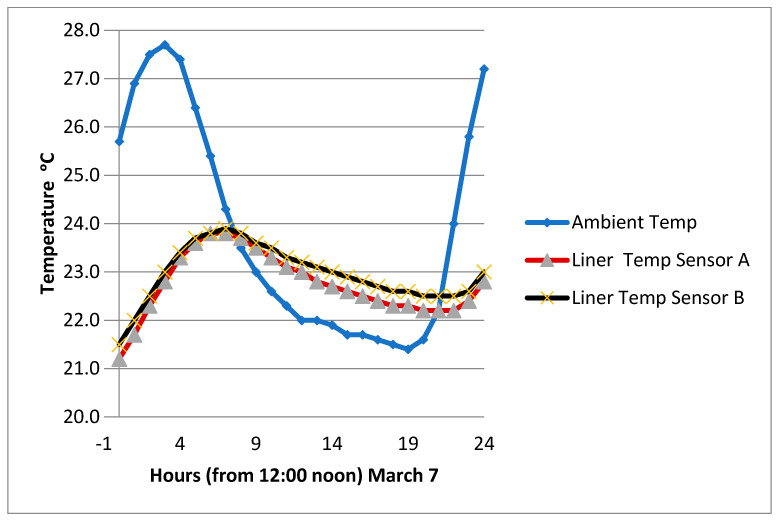
Temperature profile of 24 h of moderate weather in March.

**Figure 9:**
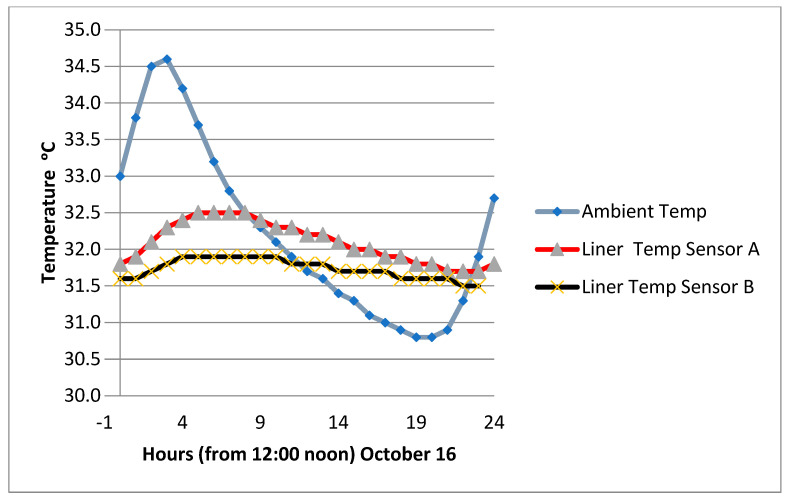
Temperature profile of 24 h of hot weather in October.

It is of interest to see what the temperature profile looks like for the ambient temperature and the temperature within the mid-section of the liner over a 24 h period. A comparison was made for two weather conditions prevailing in March and October (Figure 8 and Figure 9).

When the ambient temperature is building up in the morning, a difference of 4 °C can be observed in the mid-liner level. When a maximum temperature is reached and the weather starts to cool, the temperature in the liner continues to increase, but at a lower rate. After three hours from the maximum hot point, the temperature in the clay–sand liner became similar to the ambient temperature. In further hours, the subsurface temperature is much hotter than the ambient temperature. This is due to the fact that clay absorbs heat slowly and releases heat slowly. This property can be utilized to determine the most convenient time to use the park during hot weather conditions. Both March and October introduced the same temperature trends. As the temperature remained within 40 °C, the impact on the permittivity was small, and the calibration of the moisture measurements was not significantly affected. Knowledge of the temperature fluctuation of the near-surface soil will help with the convenience of the facility.

The response to direct watering was studied for each test. The amount of water supplied through pipes and measured in a flowmeter is given as an initial inlet reading and a final inlet reading, and the water amount in liters is computed. The date of watering and the time during which water is supplied to the grass are also recorded. The impact of watering as read in the sensor is measured as a starting vmc (volumetric water content) and the maximum vmc. The difference in vmc is computed. The time needed to reach the maximum vmc is also examined.

Table 4 summarizes 29 direct watering tests, vmc, and quantities of water supplied.

**Figure 10:**
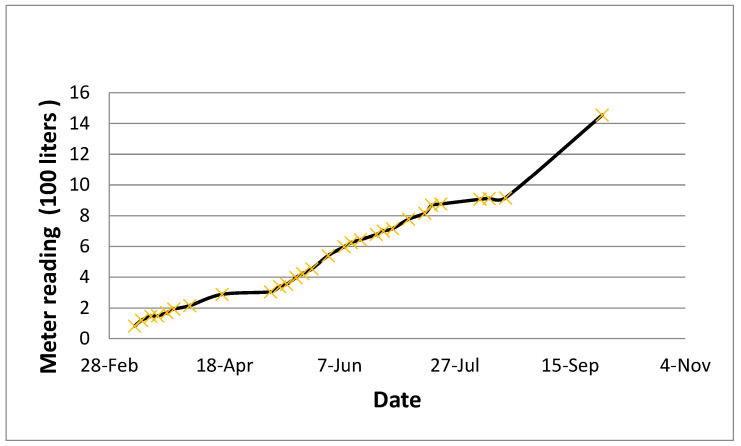
The overall water supplied to the Al-Qatif clay liner section over six months.

**Figure 11:**
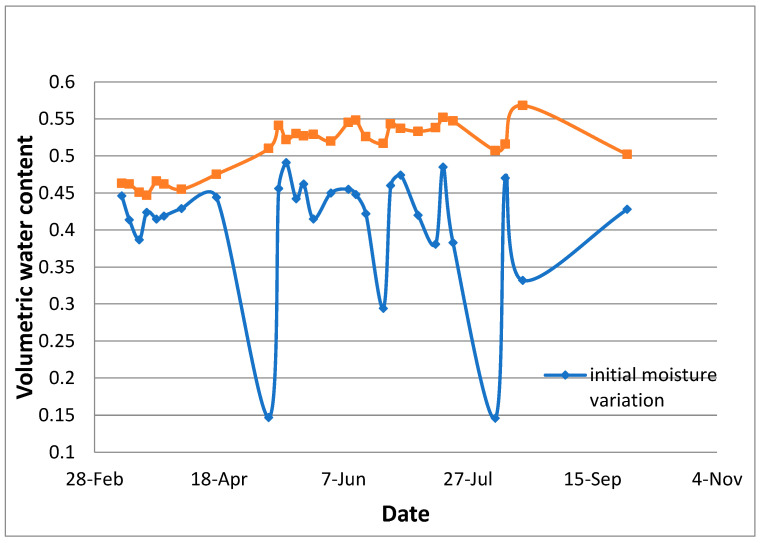
Trend of moisture variation over six months.

Figure 10 presents the overall water supplied to the Al-Qatif clay liner section over a period of six months. Figure 11 presents the starting and maximum volumetric moisture content and the general trend of moisture variation over six months.

**Figure 12:**
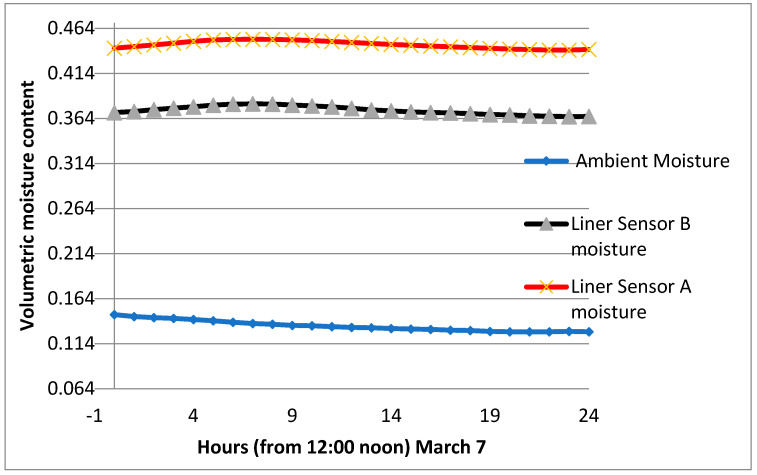
Volumetric moisture content on a non-watering day (7 March).

**Figure 13:**
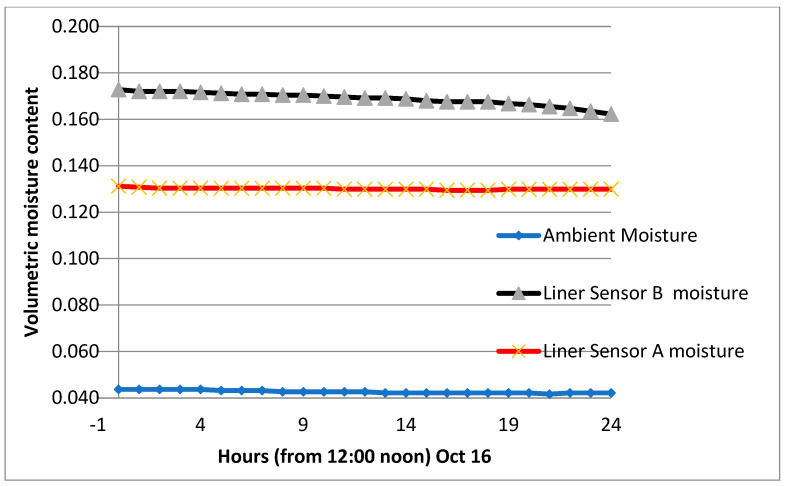
Volumetric moisture content on a non-watering day (16 October).

**Figure 14:**
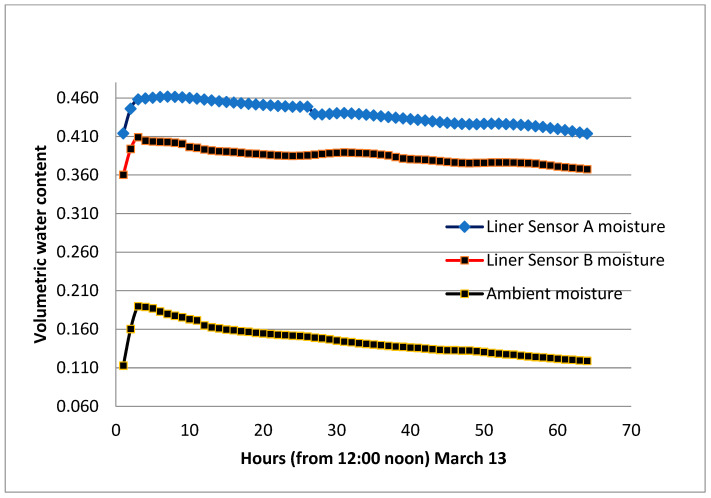
Volumetric water content response to watering (13 March).

The section was let to dry up on two occasions, and the volumetric water content was as low as 0.150. It is worth mentioning that when watering an initially dry clay liner, the gain of moisture is quick, and the maximum vmc is reached in a short time. This is attributed to the high suction created within the clay–sand mixture. The average peak of the vmc is estimated at 0.52 for the period from April to October and 0.470 in the month of March. Figure 12, Figure 13 and Figure 14 show the volumetric moisture content on selected dry and wet days.

**Figure 15:**
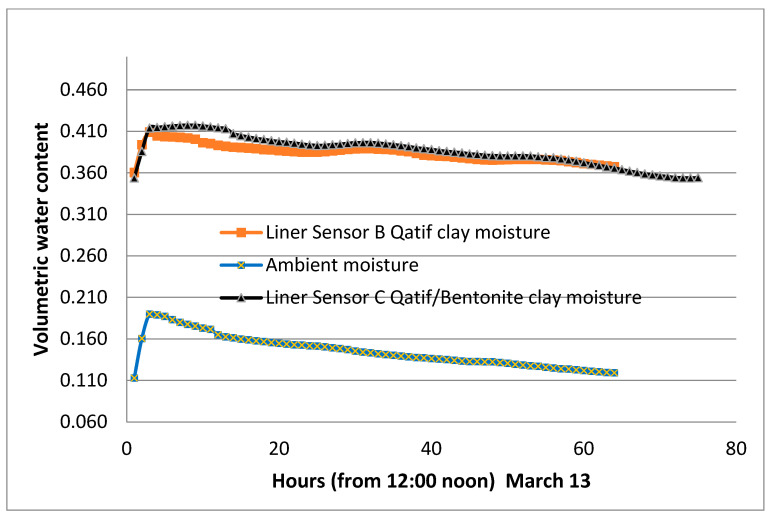
Volumetric moisture content compared with the Al-Qatif clay liner and a bentonite-enhanced Al-Qatif clay liner.

**Figure 16:**
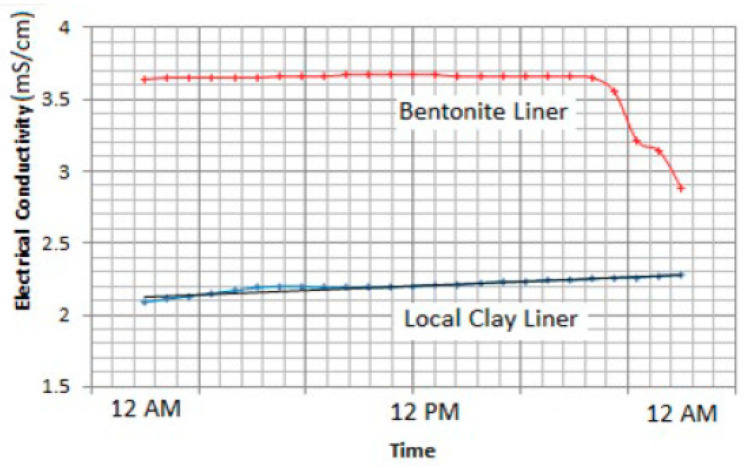
Differences in electrical conductivity between the Al-Qatif clay and the bentonite clay, as measured for a 24 h period.

For comparison purposes, another similar section consisting of 20% Al-Qatif clay enhanced by replacing one-third of the local clay with bentonite was constructed in close vicinity. This section was watered on the same dates as the main section presented in this study. Figure 15 presents the response to watering in both sections. The trend is almost similar, but the time required to dry out to the original moisture is longer for the bentonite-enhanced section. It was found to take 80 h compared to 60 h needed for the natural clay of Al-Qatif. The advantage of adding clay is the decrease in the hydraulic conductivity. The study of hydraulic conductivity is not set as an objective of this research. The composition of the two sections is different, and the bentonite is found to have different electrical conductivity, which is likely to influence the measurement of the volumetric moisture content. The variation of the electrical conductivity was presented in previous research by Dafalla et al. [33] and is shown in Figure 16.

Although soil moisture interaction is a very complex phenomenon, it has recently become easier to study and has aided researchers, geotechnical engineers, and the landscape industry in conducting more insightful studies. This is due to the advent of digital sensors that can measure electrical conductivity and moisture content, soil behavior prediction, and weather-related temperature and moisture monitoring at all times. When there is insufficient water for plants or when the soil is drying out, automatic irrigation pumps may be turned on. When used in clay–sand liners, the data obtained from these sensors can provide useful information to environmental engineers. The trends and variations in the temperature profile of clay–sand mixtures can be readily obtained. It is worth mentioning here that the output of 5TE sensors is calibrated by the manufacturer. The manufacturers conduct calibrations to make sure that the system is valid for a wide range of soils. Assessment was conducted by other researchers to validate the use of these sensors [8,34]. It is common to measure the gravimetric moisture content rather than the volumetric moisture content. According to reference [34], these sensors functioned correctly at salinities of 2.42 dS m^−1^ and temperatures of 25 °C, respectively. When the temperature and salinity of the soil were between 16 and 30 °C and 1.9 and 2.75 dS m^−1^, respectively, this sensor generally produced satisfactory findings (using data processed by manufacturer programming).

## 5. Conclusions

The use of 5TE sensors linked with a subsurface liner was found to be a promising system for saving irrigation water and monitoring the subsurface moisture content. The 5TE sensor is a TDR type, which is found to be an effective, time-saving, and early-warning tool for watering or dewatering excess water from underneath green parks.

The knowledge of the temperature fluctuation of the near-surface soil will aid in the convenient use of the facility.

The information obtained from the field log can help in determining long, relaxing periods. During the winter, green spaces with lots of clay will retain heat for a few hours and offer comparatively mild evenings. During the summer, the mixture will stay cool for a portion of the day.

Repeated watering tests confirmed a stable measuring system and reliable performance. The set-up suggested for saving irrigation water includes drainage and water collection, which needs to be addressed in future studies to select appropriate slopes and water-collection vessels. Also, a system for triggering automatic pumps needs to be investigated based on the plant’s needs and watering periods. The clay–sand liner volumetric water content can reach a value of 0.15 when no watering or wetting is conducted over a period of a few days. The electrical conductivity of the bentonite-enhanced clay–sand liner can reach a value of 3.7 mS/cm, while the Al-Qatif clay reported a maximum of 2.3 mS/cm.

Installing this sensor type is practical and can provide continuous data and information that enable quick decisions needed for watering grass and the collection of surplus water using automatic or manually controlled pumps. Future works can explore the effect of humidity on the clay–sand liner and also the hydraulic conductivity using this technology. Introduction of a control panel system may also be an option to enable automatic pumps. The data presented in this study are limited to the type of clay used but can be used for estimating the behavior of other clays with a similar mineralogy or properties.

## Figures and Tables

**Table 1:** Al-Qatif natural clay physical properties.

Property	Range
Material passing sieve number 200 (%)	>90
Liquid limit (%)	137–140
Plastic limit (%)	45–60
Plasticity index (%)	96–99
Maximum dry density (kN/m^3^)	11.5–12.0
Optimum moisture content (%)	32–40
Swell percent, ASTM D4546 [10], (%)	16–18
Swelling pressure (ASTM D4546) at a density of 12.0 kN/m^3^ (kN/m^3^)	500–800

**Table 2:** Chemical composition of Al-Qatif clay and bentonite clay.

Property	Al-Qatif Clay	Bentonite Clay
FeO_3_, %	**<0.1**	2.9
K_2_O, %	2.2	0.1
Na_2_O, %	<0.1	1.9
Al_2_O_3_, %	6.3	17
MgO, %	<0.1	4.6
SiO_2_, %	17.3	55.2
TiO_2_, %	<0.1	<0.1
CaO, %	0.9	0.9

**Table 3:** 5TE Sensor specifications [13].

Parameter	Details	
Volumetric Water Content	Range	Apparent dielectric permittivity (εa): 1 (air) to 80 (water)
	Resolution	εa: 0.1 εa (unit-less) from 1–20, <0.75 εa (unit-less) from 20–80 VWC: 0.0008 m^3^/m^3^ (0.08% VWC) from 0 to 50% VWC
	Accuracy	(εa): ±1 εa (unit-less) from 1–40 (soil range), ±15% from 40–80 (VWC): Using Topp equation: ±0.03 m^3^/m^3^ (±3% VWC) typical in mineral soils that have solution electrical conductivity < 10 dS/m Using medium-specific calibration: ±0.01–0.02 m^3^/m^3^ (± 1–2% VWC) in any porous medium
Electrical Conductivity	Range	0–23 dS/m (bulk)
	Resolution	0.01 dS/m from 0–7 dS/m, 0.05 dS/m from 7–23 dS/m
	Accuracy	±10% from 0–7 dS/m, user calibration required above 7 dS/m
Temperature	Range	−40 to 50 °C
	Resolution	0.01 °C
	Accuracy	±1 °C

**Table 4:** Summary of 29 direct watering tests, vmc, and quantities of water supplied.

Date	Initial Inlet Meter Reading	Final Inlet Meter Reading	Quantities Supplied (Liter)	Period of Watering (Minutes)	Impact on vmc			Time to Maximum vmc (Hours)
					Starting vmc	Maximum vmc	Difference in vmc	
10-March	0.8229	0.9991	176.2	20	0.446	0.463	0.017	8
13-March	1.1993	1.2662	66.9	25	0.414	0.462	0.048	6
17-March	1.4594	1.4726	13.2	20	0.387	0.451	0.064	4
20-March	1.4948	1.6718	177	20	0.424	0.447	0.023	3
24-March	1.7007	1.9246	223.9	37	0.415	0.466	0.051	3
27-March	1.9256	2.131	205.4	42	0.419	0.462	0.043	4
3-April	2.1572	2.2998	142.6	16	0.429	0.455	0.026	4
17-April	2.886	3.0482	162.2	46	0.444	0.475	0.031	5
8-May	3.0539	3.3983	344.4	37	0.147	0.51	0.363	6
12-May	3.3984	3.5432	144.8	43	0.456	0.541	0.085	5
15-May	3.5437	3.8541	310.4	55	0.491	0.522	0.031	6
19-May	3.9736	4.2353	261.7	78	0.442	0.53	0.088	3
22-May	4.2353	4.5438	308.5	53	0.462	0.527	0.065	3
26-May	4.5439	4.8146	270.7	42	0.415	0.529	0.114	6
2-June	5.4019	5.547	145.1	33	0.45	0.52	0.07	2
9-June	5.9812	6.244	262.8	33	0.455	0.545	0.09	4
12-June	6.2441	6.3527	108.6	14	0.448	0.548	0.1	4
16-June	6.4351	6.7668	331.7	39	0.422	0.526	0.104	4
23-June	6.7769	6.9994	222.5	32	0.294	0.517	0.223	5
26-June	6.9994	7.0173	17.9	35	0.46	0.543	0.083	3
30-June	7.1568	7.4156	258.8	33	0.474	0.537	0.063	3
7-July	7.7716	8.0495	277.9	23	0.42	0.533	0.113	4
14-July	8.1574	8.4565	299.1	41	0.381	0.538	0.157	5
17-July	8.6737	8.8589	185.2	32	0.485	0.552	0.067	5
21-July	8.7588	9.0656	306.8	41	0.383	0.547	0.164	9
7-August	9.0656	9.1221	56.5	32	0.146	0.507	0.361	3
11-August	9.1221	9.1579	35.8	30	0.47	0.516	0.046	3
18-August	9.1579	9.5235	365.6	59	0.332	0.568	0.236	2
29-September	14.5531	14.9682	415.1	55	0.428	0.502	0.074	4

## Data Availability

The data used to support the findings of this study are included in the introduced figures.
